# Facilitating Applications of SSVEP-Based BCIs by Within-Subject Information Transfer

**DOI:** 10.3389/fnins.2022.863359

**Published:** 2022-05-26

**Authors:** Xiaobing Liu, Bingchuan Liu, Guoya Dong, Xiaorong Gao, Yijun Wang

**Affiliations:** ^1^State Key Laboratory of Reliability and Intelligence of Electrical Equipment, Hebei Key Laboratory of Bioelectromagnetics and Neural Engineering, School of Health Sciences and Biomedical Engineering, Hebei University of Technology, Tianjin, China; ^2^Department of Biomedical Engineering, School of Medicine, Tsinghua University, Beijing, China; ^3^State Key Laboratory on Integrated Optoelectronics, Institute of Semiconductors, Chinese Academy of Sciences, Beijing, China; ^4^Chinese Institute for Brain Research, Beijing, China

**Keywords:** brain–computer interface, steady-state visual evoked potential, electroencephalography, transfer learning, cross day, cross electrode

## Abstract

The steady-state visual evoked potential based brain–computer interface (SSVEP–BCI) can provide high-speed alternative and augmentative communication in real-world applications. For individuals using a long-term BCI, within-subject (i.e., cross-day and cross-electrode) transfer learning could improve the BCI performance and reduce the calibration burden. To validate the within-subject transfer learning scheme, this study designs a 40-target SSVEP–BCI. Sixteen subjects are recruited, each of whom has performed experiments on three different days and has undergone the experiments of the SSVEP–BCIs based on the dry and wet electrodes. Several transfer directions, including the cross-day directions in parallel with the cross-electrode directions, are analyzed, and it is found that the transfer learning-based approach can maintain stable performance by zero training. Compared with the fully calibrated approaches, the transfer learning-based approach can achieve significantly better or comparable performance in different transfer directions. This result verifies that the transfer learning-based scheme is well suited for implementing a high-speed zero-training SSVEP–BCI, especially the dry electrode-based SSVEP–BCI system. A validation experiment of the cross-day wet-to-dry transfer, involving nine subjects, has shown that the average accuracy is 85.97 ± 5.60% for the wet-to-dry transfer and 77.69 ± 6.42% for the fully calibrated method with dry electrodes. By leveraging the electroencephalography data acquired on different days by different electrodes *via* transfer learning, this study lays the foundation for facilitating the long-term usage of the SSVEP–BCI and advancing the frontier of the dry electrode-based SSVEP–BCI in real-world applications.

## Introduction

A brain–computer interface (BCI) establishes an information channel between the brain and external devices without depending on a conventional spinal cord or a peripheral neuromuscular system (Lebedev and Nicolelis, 2006). The BCI technology can be used in functional recovery and replacement, providing alternative and augmentative communication for patients with motor disabilities. The steady-state visual evoked potential (SSVEP) is the brain response to periodic visual stimuli, and it has been well known for its frequency tagging attribute. The SSVEP has the same fundamental frequency and harmonic components as the visual stimulus and can maintain good time–lock and phase–lock characteristics with the stimulus signal. The high signal-to-noise ratio (SNR) profile of the SSVEP has been used to implement an SSVEP-based BCI (SSVEP–BCI), which represents a simple and easy-to-use system with a high information transfer rate (ITR) (Wang et al., 2006; Bin et al., 2009). In the SSVEP–BCI implementation process, frequency encoding and phase encoding have been the two most commonly used encoding methods. In recent research, phase coding has been integrated into frequency coding as an effective method of joint frequency and phase coding (JFPM) to increase the difference between the frequency coding targets (Chen et al., 2015b).

Apart from the encoding methods, the past two decades have witnessed rapid progress in the decoding and frequency recognition methods for the SSVEP–BCIs. Initially, the power spectral density (PSD) was employed for frequency detection to identify the SSVEP frequency by detecting the peak frequency in the Fourier transform (Wang et al., 2006). Then, the spatial filtering was used to optimize the application of the electroencephalography (EEG) data from multiple channels and reduce the common noise in channels, thereby improving the frequency recognition accuracy of the SSVEP (Wong et al., 2020b). For instance, the canonical correlation analysis (CCA) has been widely used in the SSVEP–BCI (Lin et al., 2007; Bin et al., 2009). The widespread application of the standard CCA method could be attributed to its training-free merit due to the usage of sine and cosine signals as a prior reference. Although the CCA is very efficient in frequency recognition, the recognition accuracy of the CCA is highly limited by the EEG data length due to the interference from spontaneous brain electrical activity. Recently, supervised methods, which leverage the training data for template matching, including the individual template-based CCA (IT-CCA) (Bin et al., 2011), multi-set CCA (Mset CCA) (Zhang et al., 2014), extended CCA (eCCA) (Nakanishi et al., 2014; Chen et al., 2015b), task-related component analysis (TRCA) (Nakanishi et al., 2018), and task-discriminant component analysis (TDCA) (Liu et al., 2021), have shown superior performance in SSVEP detection. The supervised methods usually compute the SSVEP template and spatial filter of different targets from the training data and then find the most similar template signal by calculating the correlation coefficient of the test data and the template after spatial filtering. Compared with the sine and cosine reference signals used in the CCA, the individual SSVEP template fully considers the subject-specific SSVEP latency, as well as differences in harmonic components; thus, improving the SSVEP detection performance. In addition, compared with the training-free method that has been used only for frequency detection, the supervised method is well suited to solve the problem of SSVEP phase detection.

The EEG has been well known for its variability associated with the anatomical structure and mental and behavioral state (Gao et al., 2014). Anatomically, the variability between subjects can be mainly attributed to the individual differences pertinent to the brain, such as the head shape, skull conductivity, and cerebral cortex folding. As for endogenous factors, the factors of blinking, psychophysiological fluctuations, and fatigue contribute to the EEG data variability among subjects (Vaughan et al., 2003). Namely, even for the same subject, EEG data from different periods or sessions can be highly variable and non-stationary, which represents the major challenge in the SSVEP-BCI. Theoretically, this challenge could be addressed by transfer learning, using a machine learning-based method to implement the BCI *via* small-sample training or zero training (Jayaram et al., 2016). The goal of transfer learning is to use the knowledge learned from an environment or previous tasks to facilitate learning in a new environment or task. The domain adaptation is a representative transfer learning-based method, which uses information-rich source–domain samples to improve the performance of the target-domain model (Pan and Yang, 2010). Two important concepts in the domain adaptation problem are as follows: (1) the source domain (*D*_*s*_) represents a domain different from the test sample but has rich supervision information, and (2) the target domain (*D*_*t*_) represents the domain where the test sample is located without labels or with only a few labels. The source and target domains often refer to the same type of task but have different distributions. Transfer learning and domain adaptation have been proven to help to mitigate the problem of SSVEP–BCI variability. For instance, Yuan et al. (2015) proposed an SSVEP detection method based on the brain–brain information transfer. Using the inherent similarity in the SSVEP signals between subjects, the SSVEP template and spatial filter information from the source subjects with a larger sample were transferred to the new target subjects; the transfer template-based CCA (ttCCA) performance was better than that of the CCA. Nakanishi et al. (2016) have found that the session-to-session transfer is effective for realizing a high-speed BCI system and zero training based on the SSVEP. Recently, a least-squares transformation (LST), which converts the training data from several existing subjects into suitable personal data and forms a supplement to personal data, was proposed (Nakanishi et al., 2020). When the number of individual trials is limited, the LST can significantly improve the SSVEP decoding performance. In addition, a domain adaptation-based method named the Align and Pool for EEG Headset Domain Adaptation (ALPHA), which shows promising performances in boosting the SSVEP–BCI system by leveraging within-day auxiliary EEG data, was developed (Liu et al., 2022). The ALPHA method can significantly outperform the ttCCA, LST, and CCA methods and facilitate the application of dry-electrode systems using the wet-electrode EEG.

For a long-term user in practical applications, e.g., an amyotrophic lateral sclerosis (ALS) patient, the daily usage of a BCI system is commonly accompanied by a library of personal EEG data. The personal data are longitudinal and show certain variability over time. Therefore, one of the main challenges is how to make full use of the within-subject EEG data acquired on the previous days or by other EEG electrodes. This also represents an important problem in real-world applications that demand the implementation of a high-speed training-free BCI system. Another motivation of this study relates to the usage of a dry electrode-based BCI system. Compared with a wet electrode-based system with the conductive paste, a dry electrode-based system is free from paste preparation and thereby promising for people’s real-life applications (Xing et al., 2018). However, the performance of dry electrodes is inferior to that of wet electrodes (Mihajlović et al., 2013). This problem can be addressed by transferring data of a wet electrode to a dry electrode to improve the performance of the dry electrode. In view of this, this study investigates the possibility of transferring within-subject data in the SSVEP–BCI by simultaneously exploiting the EEG data acquired on different days by different electrodes. In the transfer learning-based scheme, the ALPHA method is used for the same subject to perform a training-free BCI task of 40-target character spelling. The experiments conducted in this study are based on the following assumptions. A subject’s EEG data acquired on different days or by different electrodes share a common model in the feature space, e.g., the spatial pattern. By exploiting the existing EEG data and overcoming the domain shift, it is possible to extract discriminative information from the previous training data of the same subject and to apply it to a new session (day) or a new electrode. This hypothesis is verified by the offline and online experiments.

The rest of this study is stated as follows: Section “Materials and Methods” describes the materials and methods including the participants, experimental design, data collection and preprocessing, target identification, filter-bank processing, performance evaluation, and statistical analysis. Section “Results” illustrates the results of similarity analysis, cross-day wet to dry transfer, cross-day wet to wet transfer, cross-day and cross-electrode comparison, training block number effect as well as the result of validation experiment. Finally, the discussion and conclusions are given in sections “Discussion” and “Conclusion,” respectively.

## Materials and Methods

### Participants

In this study, 16 healthy subjects, among whom seven males and nine females, with an average age of 26 years and normal or corrected vision, participated in the experiments. At the beginning of the experiments, all subjects were required to read and sign the informed consent form approved by the institutional review board of Tsinghua University. Among them, nine subjects participated in the validation experiment.

### Experimental Design

**FIGURE 1:**
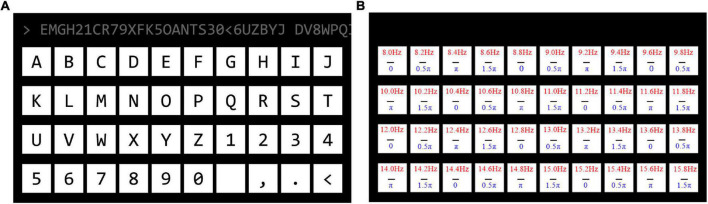
**(A)** The 40-target brain–computer interface (BCI) speller. **(B)** The frequency and the initial phase distribution of each target. The upper number indicates the frequency, and the bottom number denotes the initial phase information.

In this study, a cued spelling SSVEP–BCI experiment with a 40-target speller was designed. It was encoded by JFPM and 26 alphabets, and 10 digits and 4 symbols were presented on the screen. In the SSVEP–BCI experiment, each subject participated in both a wet-electrode headset session and a dry-electrode headset session on the same day. The tasks of the two sessions were the same, and the time interval between the two sessions was about 20–30 min. When switching headsets, the position of the electrodes was kept as close as possible, and the order of wearing the headsets was random. In each session, the subjects performed 40-target cued spelling experiments, and there was a rest period between two consecutive blocks to reduce visual fatigue. The order of stimuli was random. At the beginning of each trial, there was a 1-s red square covering the target, then all targets started to flash at the same time, and the stimulation time was 4 s. In each session, the number of blocks was six. Participants were asked to focus on the prompted target and avoid blinking during the stimulation. As shown in Figure 1, the stimulation frequency varied from 8 Hz to 15.8 Hz, and the frequency interval was 0.2 Hz, while the phase varied from zero to 1.5π, and the phase interval was 0.5π.

**FIGURE 2:**
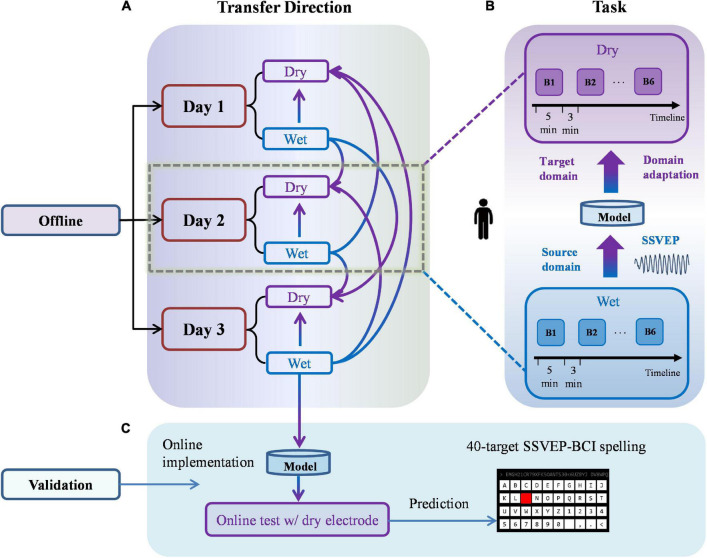
The flowchart of experiments. **(A)** The within-day wet-to-dry and cross-day wet-to-dry transfers in the offline experiment. **(B)** Specific tasks and the within-day wet-to-dry transfer in the offline experiment. **(C)** Block diagram of the validation experiment.

This study mainly used the transfer learning-based model to transfer the information across electrodes and days. Figure 2 illustrates the flowchart of the experiments and briefly introduces the transfer direction of the offline analysis. Figures 2A,B illustrate the flow of the offline experiment of a single subject. In the offline analysis, four transfer directions were investigated, namely, within-day wet-to-dry, cross-day wet-to-dry, cross-day wet-to-wet, and cross-day dry-to-dry directions. Figure 2A displays the transfer diagrams of the within-day wet-to-dry and cross-day wet-to-dry directions; the transfer diagrams of the remaining directions were similar. Figure 2B illustrates the specific experimental tasks of the two sessions and the within-day wet-to-dry transfer process. In the experiments, a wet electrode was denoted by *D*_*s*_, and a dry electrode was denoted by *D*_*t*_. The domain adaptation method was used to transfer similar information of the model trained by the wet electrode to the dry electrode. Figure 2C presents the flowchart of the validation experiment. The model was trained with the wet-electrode EEG data of day 3 and tested with the dry-electrode EEG data to conduct a 40-target SSVEP–BCI spelling experiment on a different day. The offline experiment included the data acquisition process and offline analysis of the three experiments. A detailed offline fusion analysis was conducted on the collected experimental data to select an optimal system. In the verification experiment, the selected optimal system was used to verify the results of the offline experiment to validate the feasibility of the system used in the online experiment. This study adopted only one day’s data for training in the offline analysis. The data of the closest day to the verification experiment (i.e., day 3) were used for the training in the verification experiment.

### Data Collection and Preprocessing

The data collection system and method were the same as in the study of Zhu et al. (2021). A wireless wearable NeuSenW (Neuracle, China) EEG acquisition system based on the dry and wet electrodes was used to record SSVEP data. The only difference between the dry electrode-based system and the wet electrode-based system was reflected in the electrode type. The dry-electrode headset or headband used a dry multi-pin electrode, whereas the wet electrode headset or headband used conductive paste. Eight electrodes placed in the parietal and occipital region, denoted by POz, PO3, PO4, PO5, PO6, Oz, O1, and O2, were used to record EEG data; two electrodes were placed on the forehead as reference and ground. Each data epoch started 0.4 s before the stimulus event, 0.14 s for visual latency (Chen et al., 2015a), 4 s for the stimulus, and 0.26 s after the stimulus. The collected data were downsampled to 250 Hz and then filtered by a band-pass infinite impulse response (IIR) filter to obtain signals between 6 and 90 Hz. A 50-Hz notch filter was used to remove the power frequency noise from the recorded data.

### Target Identification

The ALPHA method was compared with four other algorithms, including supervised TRCA and eCCA, ttCCA, and training-free CCA. The CCA-based target recognition algorithm with individual calibration templates was used, and templates were transferred across electrodes or days. In addition, the standard CCA, which is an unsupervised method, was used as a baseline method.

#### Standard Canonical Correlation Analysis

The CCA is a statistical method for analyzing a linear relationship between two sets of multi-dimensional variables, and it has been widely used in frequency identification of the SSVEP (Lin et al., 2007; Bin et al., 2009). Bin et al. (2009) implemented a CCA-based method in the online BCI system, which used a fixed set of nine channels in the occipital region. Since then, the CCA-based target recognition methods have gained great popularity in the SSVEP–BCI. For convenience, the CCA algorithm proposed in Bin et al. (2009) was denoted as standard CCA.

Assume that *X* ∈ *R*^*C*×*N*^ is a multi-channel EEG signal, *Y* ∈ *R*^2*N*_*h*_×*N*^ is a sine–cosine reference signal template, and *C*, *N*, *N*_*h*_, and *f*_*s*_ denote the number of EEG channels, the number of time points, the number of harmonics, and the sampling rate, respectively. Then, the sine–cosine reference signal template is given by


(1) $$Y=\left[\begin{array}{c} \begin{array}{c} \sin\left(2\,\pi \,f_{k}\,t\right)\\ \cos\left(2\,\pi \,f_{k}\,t\right) \end{array}\\ \vdots \\ \begin{array}{c} \sin\left(2\,\pi \,N_{h}\,f_{k}\,t\right)\\ \cos\left(2\,\pi \,N_{h}\,f_{k}\,t\right) \end{array} \end{array}\right],t=\left[\frac{1}{f_{s}},\ldots ,N_{P}/f_{s}\right]$$

where *f*_*k*_ is the stimulus frequency and *k* = 1, 2,…,*N*_*f*_. Here, *N*_*f*_ denotes the number of the stimuli.

In the CCA-based SSVEP detection method, the canonical correlation between a multi-channel EEG signal *X* and the reference signal *Y* is defined by


(2) $$\text{ρ}(X,Y)=\max_{w_{x},}{}_{w_{y}}\frac{E[w_{x}^{T}XY^{T}w_{y}]}{\sqrt{E[w_{x}^{T}XX^{T}w_{x}]\cdot E[w_{y}^{T}YY^{T}w_{y}]}}$$

where *w*_*x*_ *and w*_*y*_ denote the spatial filters for *X* and *Y*, respectively; the value ofρ corresponds to the largest canonical correlation between *X* and *Y*.

To identify the frequency of SSVEP in a multi-target BCI, the CCA method calculates the canonical correlation coefficients between *X* and *Y* at each stimulation frequency. The frequency of the reference signal with the largest correlation coefficient is selected as the SSVEP frequency, and it is calculated by


(3) $$f_{ta\,r\,g\,e\,t}=\arg\,\max\rho \,\left(f_{k}\right)$$

#### The Canonical Correlation Analysis With Individual Training Data

In the previous research, an extended method based on the CCA, which combines personal training data, was proposed (Nakanishi et al., 2014, 2015; Chen et al., 2015b). This method employs the CCA-based spatial filter to compute the correlation coefficient between the test set and the projected individual template, which is then used as a feature in target recognition. This method leverages important features of the individual training data to improve target recognition. In this method, first, a spatial filter $w_{\bar{x}y}$ is obtained by performing the CCA on ${\bar{X}}_{k}$ and *Y*_*f*_*k*__, which maximizes the SNR of the training set ${\bar{X}}_{k}$ of the *k*th target. In addition, by performing CCA on *X* and *Y*_*f*_*k*__, a spatial filter*w*_*xy*_ that maximizes the SNR of the test EEG data *X* is obtained. After that, the Pearson’s correlation coefficient between the test data *X* and the training data ${\bar{X}}_{k}$ is projected onto these two spatial filters, and the final correlation coefficient is calculated by


(4) $$r_{k}=\left[\begin{array}{c} r_{k,1}\\ r_{k,2}\\ r_{k,3} \end{array}\right]=\left[\begin{array}{c} r\,\left(X^{T}\,w_{\bar{x}\,y},{\bar{X}}_{k}^{T}\,w_{\bar{x}\,y}\right)\\ r\,\left(X^{T}\,w_{x\,y},{\bar{X}}_{k}^{T}\,w_{x\,y}\right)\\ r\,\left(X^{T}\,w_{x},Y_{f_{k}}^{T}\,w_{y}\right) \end{array}\right]$$

where *r*(*a*,*b*) represents the Pearson’s correlation coefficient between two one-dimensional signals, *a* and *b*.

An ensemble classifier is used to combine these three features. In fact, the weighted correlation coefficient is used as a final feature in target recognition, and it is obtained by


(5) $$\rho_{k}=\sum_{i=1}^{3}s\,i\,g\,n\,\left(r_{k,i}\right)\,\text{⋅}\,r_{k,i}^{2}$$

where *sign*(⋅) is a function used to retain identification information from the negative correlation coefficient between the test and training data or reference signals.

In this study, two training datasets were used to obtain separate models to evaluate the within-electrode and inter-electrode or inter-day variability in the SSVEP detection, and the corresponding methods were named the extended template-based CCA (eCCA) (Nakanishi et al., 2014, 2015) and the ttCCA (Yuan et al., 2015).

#### Task-Related Component Analysis

The TRCA improves the SNR of SSVEP signals by eliminating the EEG background activity, thereby enhancing the reproducibility of SSVEPs in multiple trials (Nakanishi et al., 2018). The TRCA solves the problem of recovering task-related signals from the observed EEG signals by maximizing the covariance between trials. Let us suppose *X*_*i*_ ∈ *R*^*C*×*N*^ and *X*_*j*_ ∈ *R*^*C*×*N*^ denote the *i*th and *j*th trials of a subject, respectively. The constrained optimization is given by generalized Rayleigh quotient problem as follows:


(6) w=arg maxwwTSwwTQw

where *Q* is the aggregated covariance, and it is calculated by


(7) $$Q=\sum_{i,j=1}^{N_{b}}C\,o\,v\,\left(X_{i},X_{j}\right)$$

and *S* is the inter-trial covariance, which is given by


(8) $$S=\sum_{\begin{array}{c} i,j=1\\ i\neq j \end{array}}^{N_{b}}C\,o\,v\,\left(X_{i},X_{j}\right)$$

where *N*_*b*_ is the number of training trials.

The optimal coefficient vector *w* can be obtained as an eigenvector of matrix *Q*^−1^*S* corresponding to the largest eigen-value. Then, the spatial filter *w*_*k*_1__ of the *k*_1_th stimulus frequency can be obtained. Since there are a number of individual training data samples corresponding to visual stimuli, a number of different spatial filters can be obtained. These spatial filters can be considered similar in the frequency range (Srinivasan et al., 2006; Ales and Norcia, 2009). This indicates that the recognition performance may be further improved by integrating all spatial filters. In this study, an ensemble spatial filter *W* ∈ *R*^*C*×*N*_*f*_^ was used, and it is expressed as follows:


(9) $$W=\left[w_{1},w_{2},\ldots ,w_{N_{f}}\right]$$

The correlation coefficient *r*_*k*_ between the test trial *X*_*t*_ ∉ *R*^*C*×*N*^ and the average training data ${\bar{X}}_{k}$ can be calculated by


(10) $$r_{k}=\rho \,\left(X_{t}^{T}\,W,{\bar{X}}_{k}^{T}\,W\right)$$

Then, the target frequency *f*_*t*_ can be obtained by


(11) $$f_{t}=\max_{k}\left(r_{k}\right),k=1,\ldots ,N_{f}$$

#### The ALPHA Algorithm

The ALPHA algorithm mainly includes the following three parts: Subspace decomposition, subspace alignment, and subspace pooling. By analyzing the multi-dimensional data in the low-dimensional space, the key information and features in the source domain are transferred to the target domain through decomposition, alignment, and pooling. This method leverages domain adaptation, and it belongs to the category of transfer learning. The main idea is to map data features of different domains (e.g., different datasets) to the same feature space so that data from the other domains can be used to enhance target domain. In the following, the subspace alignment process is briefly introduced.

##### Align Spatial Pattern

The goal of subspace alignment is to align *D*_*s*_ and *D*_*t*_ to the same subspace to bridge the gap between these two domains. Two types of subspace alignment are used in the ALPHA, namely, Align Spatial Pattern (ASP) and Align Covariance (AC). In the ASP, the spatial filters *W*_*1*_ and *W*_2_correspond to *D*_*s*_ and *D*_*t*_, respectively. Then, the corresponding spatial pattern can be calculated by


(12) $$A_{1}=W_{1}^{-T},A_{2}=W_{2}^{-T}$$

In this study, it assumes that *D*_*s*_ and *D*_*t*_ have the same basic spatial pattern after rotation. Therefore, the ASP method seeks to transform the matrix *P* orthogonally to the estimated spatial pattern, which yields to the following optimization problem:


(13) $${\text{minimize}}_{P}\,{||A_{1}-A_{2}\,P^{T}||}_{F}^{2},P\,P^{T}=I$$

The solution of the Procrustes problem is given by


(14) $$P=U\,V^{T}$$

where *U* and *V* are the left and right singular vectors of $A_{1}^{T}\,A_{2}$.

Then, the transformation equation of spatial filter*W*_2_ is given by


(15) $$W_{1}^{A\,S\,P}=W_{2}\,P^{T}$$

Through the ASP transformation, the characteristic information in *W*_*2*_ can be transferred to$W_{1}^{A\,S\,P}$.

##### Align Covariance

In addition to changes in spatial patterns, this study assumes that domain gaps also exist in covariance changes. Therefore, to reduce the covariance shift, the covariance between *D*_*s*_ and *D*_*t*_ distributions should be aligned. Here, we use a method based on correlation alignment (Sun et al., 2017) to achieve the goal. Also, CORAL finds a linear transformation *Q* to minimize the distance between the second-order statistics of *D*_*s*_ and *D*_*t*_.


(16) $${\text{minimize}}_{Q}\,{||Q^{T}\,C_{s}\,Q-C_{t}||}_{F}^{2}$$

Then, *C*_*s*_ and *C*_*t*_ can be, respectively, obtained by


(17) $$C_{s}^{i}=\frac{1}{N_{t}\,N-1}\,X_{m}^{i}\,C_{N_{t}\,N}\,X_{m}^{i\,T},i=1,\cdots ,N_{f}$$

(18) $$C_{t}=\frac{1}{N-1}\,X_{t}\,C_{N}\,X_{t}^{T}$$

where $C_{\text{n}}=I-\frac{1}{\text{n}}\,11^{\text{T}}$ is a centering matrix, $X_{m}^{i}\,$ is the concatenation of trials *X*_*s*_ corresponding to the *i* target, and *N*_*t*_ denotes the number of training blocks.

Next, the optimal solution to Eq. (16) is given by


(19) $$Q=C_{s}^{-\frac{1}{2}}\,C_{t}^{\frac{1}{2}}$$

For the spatial filter after AC, *W*_*X*_ can be expressed as


(20) $$W_{X}^{A\,C}=Q\,W_{X}$$

For a detailed introduction in the ALPHA, see Liu et al. (2022).

#### Filter–Bank Processing

The filter–bank technique decomposes the SSVEP into sub-band components first and then extracts the high-SNR independent information embedded in the harmonic components. Therefore, the filter–bank technique can facilitate target classification (Chen et al., 2015a). In this study, the lower and upper cut-off frequencies of sub-band *S*_*b*_ were set to (*S*_*b*_×8)−2 Hz and 90 Hz, and *S*_*b*_ was set up to five. The correlation coefficients obtained for all sub-bands were weighted to obtain the final target recognition as follows:


(21) $$\tilde{\rho_{k}}=\sum_{n=1}^{S_{b}}v\,\left(n\right)\,\text{⋅}\,{\left(\rho_{k}^{n}\right)}^{2}$$

where*v*(*n*) = *n*^−*a*^ + *b*, *n* ∈ **[**1 *S*_*b*_**]** is the weight function. According to Chen et al. (2015a), *a* was set to 1.25 and *b* was set to 0.25.

The filter–bank analysis method could extract meaningful features from different sub-band components of the EEG signal and improve the SSVEP recognition accuracy. In the results of the offline analysis and validation experiment, the filter–bank analysis method was applied to all target recognition methods. In this study, the CCA was the traditional filter–bank CCA (FBCCA).

### Performance Evaluation

The performance was evaluated using two evaluation metrics, the target identification accuracy (%) and ITR (bits/min). The ITR is calculated by


(22) $$I\,T\,R=\left(\log_{2}N_{f}+P\,\log_{2}P+\left(1-P\right)\,\log_{2}\left[\frac{1-P}{N_{f}-1}\right]\right)\times \left(\frac{60}{T}\right)$$

where *N*_*f*_ is the number of visual stimuli, *P* is the target identification accuracy, and *T* is the average selection time.

In this study, the gaze shifting time was set to 1 s. When calculating the ITR, 1 s was added to *T*.

For the TRCA and eCCA, a sixfold cross-validation was used. In each fold, five blocks were used for training, and one block was used for the testing. The number of test trials was identical for all the methods. For the ALPHA and ttCCA, all six blocks of within-day-cross-electrode or cross-day data were used for the training. The within-day wet-to-dry was the average of three experiments. Cross-day wet-to-dry (or wet-to-wet or dry-to-dry) was the average of six directions in three experiments, e.g., day1-wet to day2-dry, day2-wet to day1-dry, day1-wet to day3-dry, day3-wet to day1-dry, day2-wet to day3-dry and day3-wet to day2-dry. The average accuracies and ITR of the five methods (CCA, ttCCA, eCCA, TRCA, and ALPHA) were compared for different data lengths. All analyses in this study used data from the same subject.

### Statistical Analysis

To compare the methods, the two-way repeated measures analysis of variance (ANOVA) was performed to determine whether there was a statistically significant relationship between the method performance and the data length. The Greenhouse–Geisser correction was applied due to the violation of sphericity. There was no analysis of multiple comparisons. In the comparison of the methods or sets, the planned paired *t*-test was used to evaluate the statistical significance. The planned paired *t*-test was performed by the test function in MATLAB. The paired sample *t*-test was used to make the test decision for the original hypothesis, which assumed that the mean of the paired difference between two datasets was equal to zero and the variance was unknown.

## Results

As shown in Table 1, the transfer direction from two aspects: session (within-day or cross-day) and electrode (wet-to-wet, wet-to-dry or dry-to-dry) was divided into several types in this study. There were no transfer in within-day wet and within-day dry. The impedance value was recorded before each block. The impedance on day 1, day 2, and day 3 was stable across days, and the average impedances for the dry and wet electrodes were about 363.94 and 6.64kΩ, respectively. As shown in Table 2, there was no significant difference in impedance between any 2 of the 3 days, with all *p* > 0.05.

**TABLE 1:** The type of transfer direction.

Type	Session	Electrode type
a	Within-day	Wet
b		Dry
c		Wet to dry
d	Cross-day	Wet to wet
e		Dry to dry
f		Wet to dry

**TABLE 2:** The statistical significance of *p*-value in the impedance comparison of experiments.

Cross-day	Dry	Wet
Day 1–day 2	0.92 (345.21 kΩ *vs*. 349.41 kΩ)	0.42 (6.27 kΩ *vs.* 7.02 kΩ)
Day 2–day 3	0.30 (349.41 kΩ *vs.* 397.21 kΩ)	0.38 (7.02 kΩ *vs.* 6.64 kΩ)
Day 1–day 3	0.33 (345.21 kΩ *vs.* 397.21 kΩ)	0.65 (6.27 kΩ *vs.* 6.64 kΩ)

### Similarity Analysis Results

**FIGURE 3:**
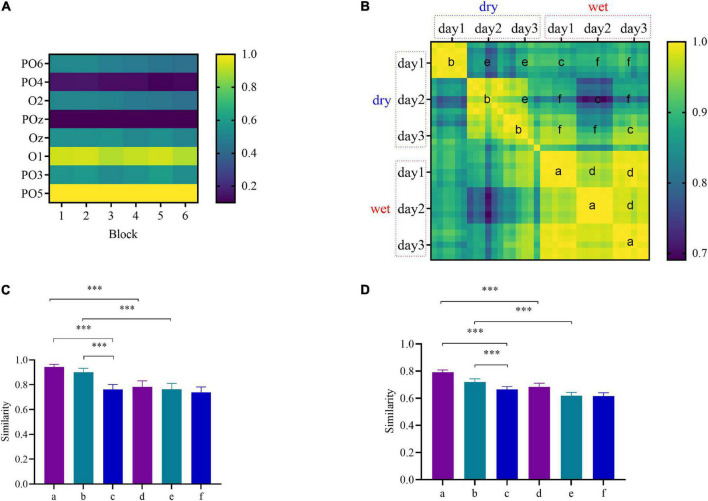
**(A)** The multi-channel steady-state visual evoked potential (SSVEP) responses over six blocks on day 1 for the wet electrodes of subject S6; the color bar indicates the normalized amplitude. **(B)** The correlation coefficient matrix between any two blocks of subject S6 for four types; the color bar indicates the similarity. **(C)** The overall average similarity of the topographical maps of all subjects. **(D)** The overall average similarity of the covariance matrices of all subjects. The asterisks indicate a difference in the paired *t*-test results (*p < 0.05, **p < 0.01, and ***p < 0.001).

According to the previous studies (Wong et al., 2020a,2021b), subjects’ brain topography has a similar spatial pattern at all stimulation frequencies. Figures 3A,B display the SSVEP responses and similarity of a representative subject (Subject S6), respectively. Each column in Figure 3A indicates the SSVEP responses (from occipital) for a specified block, where the SSVEP amplitudes denote the averaged values of 40 stimulation frequencies. Each row represents the electrode’s location, and the color indicates the normalized amplitude value. The similarity value was calculated using the correlation coefficient between any two blocks. As shown in Figure 3B, the correlation coefficient matrix of the 6× 6 square represented by each letter was between the same or different session and electrode. For each subject, the brain topographical mapping similarity was divided into the following four types: within-day-within-electrode (WDWE), within-day-cross-electrode (WDCE), cross-day-within-electrode (CDWE), and cross-day-cross-electrode (CDCE).

As illustrated in Figure 3B, for subject S6, the average correlation coefficient between the brain mappings corresponding to different types was as follows. For the WDWE, for the dry electrodes, the average correlation coefficient was 0.98, and for the wet electrodes, the average correlation coefficient was 0.99. For the WDCE, the average correlation coefficient was 0.87. For the CDWE, for the dry electrodes, the average correlation coefficient was 0.84, and for the wet electrodes, the average correlation coefficient was 0.98. For the CDCE, the average correlation coefficient was 0.88. As shown in Figure 3C, the overall average similarity over all subjects was as follows. For the WDWE, for the dry electrodes, the overall average similarity was 0.90 ± 0.03, and for the wet electrodes, the overall average similarity was 0.94 ± 0.02. For the WDCE, the overall average similarity was 0.76 ± 0.04. For the CDWE, for the dry electrodes, the overall average similarity was 0.76 ± 0.05, and for the wet electrodes, the overall average similarity was 0.78 ± 0.05. For the CDCE, the overall average similarity was 0.74 ± 0.04. The similarity results showed that the transfer difficulty of the CDCE was greater than that of the CDWE. The similarity between the wet electrodes was higher than that between the dry electrodes, which indicated that the signal collected by the wet electrodes was relatively stable.

In addition, the covariance matrix was computed for each trial, and the similarity of the covariance matrix of the four types, i.e., WDWE, WDCE, CDWE, and CDCE was compared. As shown in Figure 3D, the overall average similarity over all subjects was as follows. For the WDWE, for the wet electrodes, the overall average similarity was 0.79 ± 0.02, and for the dry electrodes, the overall average similarity was 0.72 ± 0.02. For the WDCE, the overall average similarity was 0.66 ± 0.02. For the CDWE, for the wet electrodes, the overall average similarity was 0.68 ± 0.03, and for the dry electrodes, the overall average similarity was 0.62 ± 0.02. For the CDCE, the overall average similarity was 0.62 ± 0.02. The similarity results were roughly the same as the brain topographic map results. Generally, the within-day similarity was significantly higher than the cross-day similarity regardless of the electrode type (*p* < 0.001). Both cross-electrode and the cross-day have impact on the transfer of information.

### Cross-Day Wet-to-Dry Transfer Experiment Results

**FIGURE 4:**
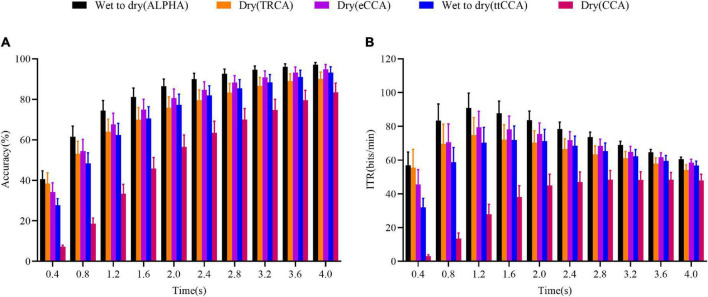
Comparison of the accuracy and ITR results of the five methods in the cross-day wet-to-dry transfer experiment. **(A)** Accuracy. **(B)** ITR value. The horizontal axis represents the data length; the error bars indicate standard errors.

The performances of the transfer learning-based methods (ALPHA and ttCCA), fully calibrated methods (TRCA and eCCA), and a training-free method (CCA) are presented in Figure 4. The two-way ANOVA showed that there was a significant interaction between the methods’ performances and data length; *F*(1.765,26.47) = 11.37,*p* < 0.001 for accuracy, and *F*(1.778,26.67) = 15.67,*p* < 0.001 for the ITR value. As shown in Figure 4A, the ALPHA outperformed the ttCCA and CCA for all data lengths (*p* < 0.05, planned paired *t*-test) and eCCA (*p* < 0.05) for all data lengths except for the data length of 4 s (*p* = 0.08). There was no significant difference in performance between the TRCA and eCCA (*p* > 0.05) except for the data length of 0.4 s (*p* = 0.02). Compared with the TRCA, the ALPHA significantly improved the accuracy except for the data length of 0.4 s (ALPHA *vs.* TRCA: 40.56 ± 4.07% *vs.* 38.48 ± 5.28%, *p* = 0.41). The performance of the eCCA was significantly better than that of the ttCCA for all data lengths (*p* < 0.05). This was because the model of eCCA was the WDWE type, while that of the ttCCA was the CDCE type. Overall, the performance of the ALPHA was better than those of the TRCA and eCCA. For all methods, the ITR changing trends with the data length were consistent with the changing trends of the accuracy. When the time length was 1.2 s, the ITR values were as follows: The ALPHA had the ITR value of 90.92 ± 8.81 bits/min; the TRCA had the ITR value of 74.85 ± 10.21 bits/min; the eCCA had the ITR value of 79.39 ± 9.52 bits/min; the ttCCA had the ITR value of 70.30 ± 9.06 bits/min; and the CCA had the ITR value of 27.94 ± 5.84 bits/min. In general, the ALPHA improved the ITR significantly. The results showed that the ALPHA in the cross-day wet-to-dry transfer performed significantly better than the fully calibrated TRCA and eCCA, indicating that ALPHA could achieve cross-day and cross-electrode recalibration-free transfer learning.

### Cross-Day Wet-to-Wet Transfer Experiment Results

Next, the cross-day wet-to-wet transfer experiment was conducted. Compared with the dry electrodes, the EEG from the wet electrodes had relatively stable signals and less noise. The cross-day wet-to-wet transfer also can reflect the advantages of the ALPHA.

**FIGURE 5:**
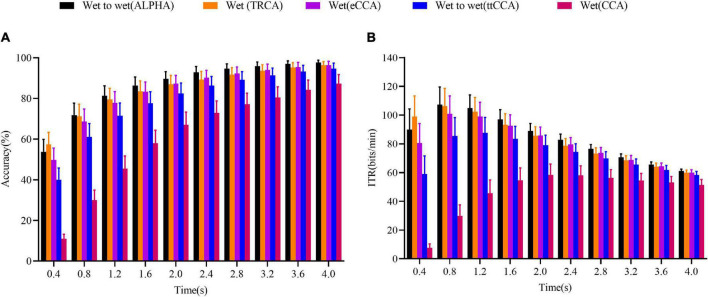
Comparison of the accuracy and ITR results of the five methods in the cross-day wet-to-wet transfer experiment. **(A)** Accuracy. **(B)** ITR value. The horizontal axis represents the data length; the error bars indicate standard errors.

The results of the cross-day wet-to-wet transfer are shown in Figure 5, where the results of accuracy and ITR at all data lengths are presented. The two-way ANOVA’s results showed that there was a significant interaction between the method performance and data length for both accuracy and the ITR value; *F*(1.809,27.14) = 16.39,*p* < 0.001 in accuracy (Figure 5A) and *F*(1.854,27.81) = 21.29,*p* < 0.001 in ITR (Figure 5B). The ALPHA significantly improved the accuracy compared with the ttCCA and CCA for all data lengths (*p* < 0.05). There was no significant difference in accuracy between the ALPHA and eCCA for all data length (*p* > 0.05). Also, there was no significant difference in accuracy between the ALPHA and TRCA except for the data length of 0.4 s (ALPHA *vs.* TRCA: 44.74 ± 5.95% *vs.* 50.28 ± 5.86%, *p* = 0.03 in the planned paired *t*-test). When the data length was relatively short (i.e., 0.4, 0.8, or 1.2 s), the TRCA performance was significantly better than that of the eCCA, and there was no significant difference between them with the increase in the data length. The performance of the eCCA was significantly better than that of the ttCCA for all data lengths (*p* < 0.05). When the data length was 1.2 s, the ITR values of the methods were as follows. The ALPHA had the ITR value of 104.88 ± 9.12 bits/min; the TRCA had the ITR value of 102.37 ± 9.84 bits/min; the eCCA had the ITR value of 99.07 ± 9.82 bits/min; the ttCCA had the ITR value of 87.66 ± 10.71 bits/min; and the CCA had the ITR value of 45.65 ± 9.16 bits/min. The difference in the ITR value between the methods for different data lengths was roughly the same as the difference in the accuracy.

### Cross-Day and Cross-Electrode Transfer Comparison Results

From a practical point of view, dry electrodes are more convenient and easier to use than wet electrodes. However, the dry electrodes have the disadvantages of high impedance and much noise, which leads to poor performance. Therefore, the model trained with the wet-electrode data was applied to the dry-electrode data to improve the performance of the dry electrodes and promote the application of the SSVEP–BCI in people’s lives. The performances of the within-day wet-to-dry, cross-day wet-to-dry, and cross-day dry-to-dry transfers were compared. The within-day wet-to-dry transfer result was the average result of day 1, day 2, and day 3, and the results of the cross-day wet-to-dry and cross-day dry-to-dry transfers were the average results of the results in the six transfer directions.

**FIGURE 6:**
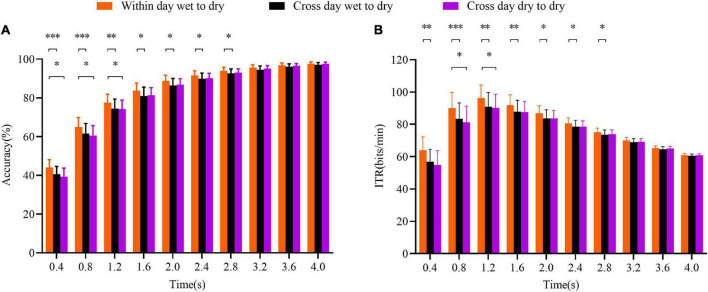
Performance comparison of the ALPHA method for different transfer types. **(A)** Accuracy. **(B)** ITR value. The cross-day transfer corresponds to the time dimension, while the wet-to-dry and dry-to-dry transfers correspond to the electrode dimension. The error bars indicate standard errors. The asterisks indicate a difference in the paired *t*-test results (*p < 0.05, **p < 0.01, and ***p < 0.001).

As shown in Figure 6, the overall performance of the within-day wet-to-dry transfer was better than that of the cross-day wet-to-dry transfer. When the data length was relatively short, the performance of the within-day wet-to-dry transfer was significantly better than those of the cross-day wet-to-dry and cross-day dry-to-dry transfer, with *p* < 0.05. As the data length increased, there was no significant difference between the three transfer types. It is worth mentioning that there was no significant difference in performance between the cross-day wet-to-dry and cross-day dry-to-dry transfers with the data length, *p* > 0.05. The stability of the wet-electrode data was better than that of the dry-electrode data, and transferring two dimensions was more difficult than transferring one dimension. The cross-day wet-to-dry transfer had two dimensions to transfer, i.e., time and electrode, which decreased the performance compared to the within-day wet-to-dry transfer. The difference in the ITR value between the three transfer types was consistent with that of the accuracy. The statistical results of the *t*-test are presented in Figure 6.

**FIGURE 7:**
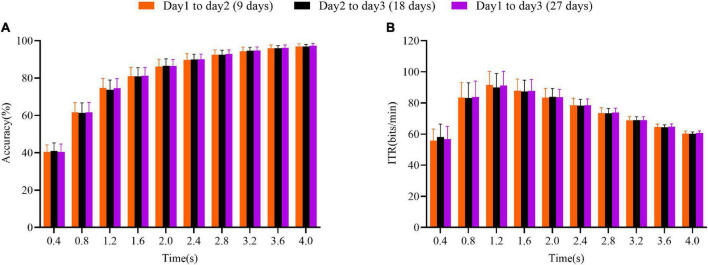
The influence of the number of days between experiments on the ALPHA performance. **(A)** Accuracy. **(B)** ITR value. The presented results denote the average results of the mutual transfer between two days. The error bars indicate standard errors.

Next, it was analyzed whether the length of the cross-day wet-to-dry transfer had an effect on the ALPHA algorithm’s performance. The analysis results are shown in Figure 7. In the experiments, the average interval between day 1 and day 2 for all subjects was approximately 9 days, and the interval between day 2 and day 3 was approximately 18 days. Statistical analysis results showed that there was no significant difference in performance with the increase of the data length (*p* > 0.05, planned paired *t*-test). The result indicated that the cross-day time of about 1 month had no effect on the ALPHA performance. Although it was almost impossible to place the electrodes in exactly the same position on different days, the spatial filtering and feature extraction of the ALPHA could improve the template matching performance. As for the cross-day wet-to-dry transfer, in order to confirm the feasibility of the transfer effect of ALPHA, we carried out a series of feature visual analysis before and after ALPHA in the Supplementary Material. Compared to the dry-TRCA (the TRCA with the dry electrodes), the ALPHA could maintain stable performance by zero training across days.

### Training Block Number Effect

**FIGURE 8:**
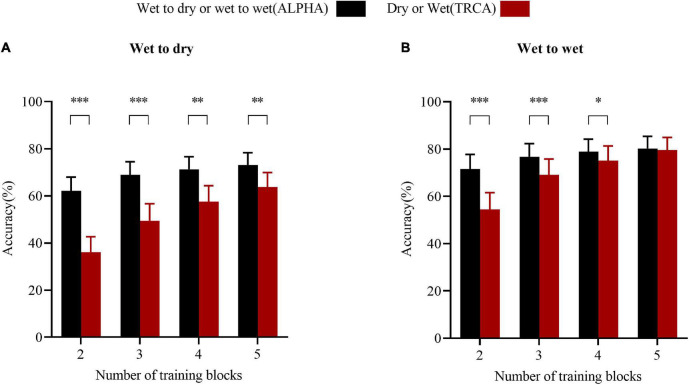
The performance comparison of the ALPHA and TRCA methods for different numbers of training blocks. **(A)** Accuracy. **(B)** Accuracy. The error bars indicate standard errors. The asterisks indicate a difference in the paired *t*-test results (*p < 0.05, **p < 0.01, and ***p < 0.001).

An important parameter of reducing training time is to reduce the number of training blocks. To reduce the impact between the blocks, cross-validation was performed. For instance, consider the number of training blocks was two. For TRCA, two blocks were randomly selected for training and the remaining blocks were used for the testing every time, and performed the same process six times in total. For ALPHA, two blocks from the wet electrodes were randomly selected for training and six blocks from the wet electrodes or dry electrodes were used for the testing every time, and performed the same process six times in total. The results showed that as the number of individual training blocks decreased, ALPHA could be robust enough to detect the SSVEP with sufficient accuracy. The average accuracy results of the ALPHA and TRCA methods for different numbers of individual training blocks in the cross-day wet-to-dry and cross-day wet-to-wet transfer experiments are presented in Figure 8. In these experiments, the data length was 1.2 s. According to the results, with the increase in the training block number, the accuracy improved.

For the wet-to-dry transfer, the ALPHA significantly improved the detection accuracy; the results of ALPHA *vs.* TRCA were as follows: In the two-block experiment, 62.08 ± 5.87% *vs.* 36.06 ± 6.62%; in the three-block experiment, 68.92 ± 5.55% *vs.* 49.47 ± 7.16%; in the four-block experiment, 71.27 ± 5.38% *vs.* 57.50 ± 6.83%; and in the five-block experiment, 73.12 ± 5.19% *vs.* 63.72 ± 6.18%, respectively. For the wet-to-wet transfer, when the number of training blocks was small, the performance of the ALPHA was better than that of the TRCA. With the increase in the training block number, there was no significant difference between the ALPHA and TRCA methods. The results of ALPHA *vs.* TRCA were as follows: In the two-block experiment, 71.45 ± 6.25% *vs.* 54.48 ± 7.08%; in the three-block experiment, 76.72 ± 5.61% *vs.* 69.04 ± 6.71%; in the four-block experiment, 78.83 ± 5.32% *vs.* 75.10 ± 6.15%; and in the five-block experiment, 80.22 ± 5.10% *vs.* 79.56 ± 5.37%, respectively. It is worth noting that in the cross-day wet-to-dry transfer experiment, the performance of the ALPHA for two training blocks was similar to that of the TRCA for five training blocks; namely, the performances of the ALPHA and TRCA methods were 62.08 ± 5.87% and 63.72 ± 6.18%, respectively. With the increase in the training block number, the performances of the ALPHA and TRCA methods could be improved. Moreover, the ALPHA method generally performed better than the TRCA method in both wet-to-dry and wet-to-wet transfer experiments.

### Validation Experiment and Results

According to the offline analysis result, this study chose the cross-day wet-to-dry transfer system for the online verification experiment based on practicability and performance. The stimulation time of the validation experiment was 1.2 s. The wet electrode data of the third experiment were used for the training, and the validation experiments were performed with the dry electrodes a few days after the third experiment. Nine subjects with good offline experimental results participated in the validation experiment. The average interval between the verification experiment and the day 3 experiment was approximately 54 days. The results of the nine subjects are given in Table 3, and according to the results, the performance of the ALPHA was better than that of the dry-TRCA. The results of ALPHA and dry-TRCA were, respectively, as follows: Regarding the accuracy, 85.97 ± 5.60% and 77.69 ± 6.42%, and *p* = 0.03; regarding the ITR value, 112.84 ± 10.60 bits/min and 96.56 ± 12.22 bits/min, and *p* = 0.03. For the ALPHA and TRCA methods, there was no significant difference between the validation and offline results in terms of the accuracy; namely, the ALPHA had *p* = 0.97, and the TRCA had *p* = 0.73. The ITR results of the two methods were consistent with those of the accuracy. For subjects S1 and S9, the results of the validation experiment were much lower than those of the offline experiment for both ALPHA and TRCA. This could be because the impedance of the verification experiment was higher than that of the offline experiment. Thus, if the impedance of the dry electrode could be further reduced, the performances of the ALPHA and dry-TRCA methods would be improved (Zhu et al., 2021).

**TABLE 3:** Result comparison of the validation experiment and offline analysis.

	Accuracy (%)/ITR (bits/min)			
Subject	ALPHA (validation)	TRCA (validation)	ALPHA (offline)	TRCA (offline)
S1	75.83/88.72	55.83/55.00	86.81/114.42	87.08/112.16
S2	93.33/126.15	80.00/97.29	87.01/112.03	85.28/107.96
S3	98.33/139.67	99.17/142.41	92.71/126.62	88.47/117.75
S4	96.67/134.90	90.42/119.27	88.89/116.64	71.52/84.41
S5	98.75/141.04	97.50/137.40	92.85/125.98	97.36/137.40
S6	90.00/118.55	67.08/72.85	96.94/135.84	89.86/119.99
S7	87.08/111.91	75.00/87.26	77.78/93.46	30.28/21.60
S8	88.33/114.48	90.83/120.30	84.79/107.13	74.72/88.03
S9	45.42/40.10	43.33/37.25	67.22/74.37	50.00/49.10
**Mean**	**85.97/112.84**	**77.69/96.56**	**86.11/111.50**	**74.95/93.16**
**SD**	**5.60/10.60**	**6.42/12.22**	**3.00/6.21**	**7.26/12.38**

*Bold values are used to emphasize the average level of all subjects.*

## Discussion

**FIGURE 9:**
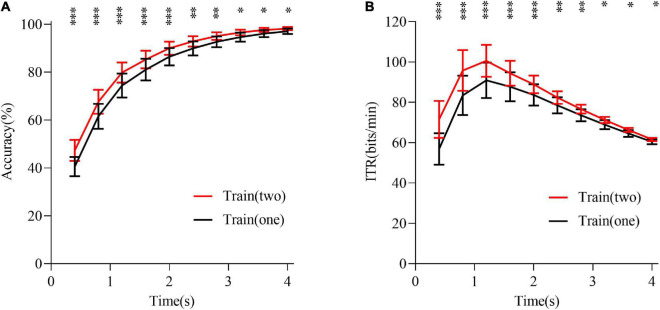
Performance comparison of the Train (one) and Train (two). **(A)** Accuracy. **(B)** ITR value. Train (one) means that data of one of the three experiments were used for training, and data of the other two experiments were used for testing; there were six cases in total. Train (two) means that data of two of the three experiments were used for training, and data of the remaining experiment were used for testing; there were three cases in total. The asterisks indicate a difference between Train (one) and Train (two) in the paired *t*-test (*p < 0.05, **p < 0.01, and ***p < 0.001).

The benefit of leveraging training data across 2 days (session) in the offline analysis was further investigated. In the cross-validation experiment, data of day 1, day 2, and day 3 were used, namely, data of 2 days were used for the training, and data of the remaining day were used for the testing. The training data were the average data of the 2 days. The results of the wet-to-dry transfer experiment are shown in Figure 9, where it can be seen that the training performance for two-day data was significantly better than that of the one-day data, with *p* < 0.05. The results demonstrated an increasing benefit of transfer learning as more EEG data were available in the daily usage of BCI. Therefore, for the long-term BCI applications, the wet electrodes could be more convenient for performance calibration and offline validation at the beginning, but then it should be gradually transitioned to the dry electrodes, thus forming a personal database containing the EEG data from both dry and wet electrodes. This personal database could be exploited to implement a high-speed zero-training SSVEP–BCI in the future.

As for transfer learning, this study extends the wet-to-dry transfer (Liu et al., 2022) to a cross-day scenario and verifies the effectiveness of the ALPHA method in the cross-day and cross-electrode transfers for the 40-target SSVEP–BCIs. The results show that the ALPHA can extract similar information of the same subject and suppress the variability across days and electrodes. In the ALPHA method, there are three essential steps for feature extraction and domain adaptation, i.e., subspace decomposition, subspace alignment, and subspace pooling. Subspace alignment aligns information in the source and target domains, and subspace pooling performs feature fusion. For instance, He and Wu (He and Wu, 2020) applied the subspace alignment to the motor imagery paradigm by aligning the EEG of different subjects in the Euclidean space and then applying it to new subjects. Chen et al. (2019) proposed a progressive feature alignment network, which gradually and effectively aligns cross-domain discriminant features according to the intra-class changes in the target domain.

Apart from leveraging information across electrodes and sessions, transfer learning could also be used to exploit common information of SSVEP response across subjects. For instance, (Tanaka, 2020) used the multivariate method to extract repeated components between the trials and subjects. This method can maximize not only the trial repeatability of a single subject but also the similarity between a group of subjects. Wang et al., 2021 proposed a new inter- and intra-subject maximum correlation (IISMC) method to enhance the robustness of SSVEP recognition using the similarity between and within subjects. Yuan et al. (2015) and Wong et al. (2020c) transferred common information between the subjects, and a good classification performance was achieved when the number of training trials was reduced.

In this study, the scope of interest is limited to the within-subject variability, and the data obtained by different electrodes in different periods from the same subject are used to implement a zero-training BCI. For the purpose of a plug-and-play BCI system, the cross-subject variability should be considered, and the gap between subjects in the domain shift should be bridged. Since the data transfer across the subjects is challenging, in the future work, more methodological approaches could be used to improve the practical application of the SSVEP–BCI by mitigating the variability across electrodes, sessions, and subjects.

For the cross-subject transfer, online adaptation is a promising method to reduce new subjects’ training time and to avoid calibration (Spüler et al., 2012; Waytowich et al., 2016; Wong et al., 2021a). Our future work is to develop the domain adaptation of the ALPHA method to the adaptive online method of new subjects. By iteratively updating the training model of multiple subjects, a higher performance could be achieved for new subjects. However, it is crucial to use appropriate strategies to update and iterate the model so as to approximate a new subject’s model with the adaptive process. An unsupervised online adaptive method could ensure the development of a high-performance online BCI system. Therefore, in the future, the ALPHA’s feature extraction methods could be applied to the code-modulated-VEP (c-VEP)-based BCI system since both c-VEP and the SSVEP have common feature information (Thielen et al., 2021).

## Conclusion

To make an SSVEP–BCI spelling system more convenient for real-life applications, this study leverages within-subject information in the same task by transferring it across days and electrodes. By analyzing different transfer directions, the feasibility of the within-subject transfer is verified, and the stable performance of transfer learning across days is demonstrated. The experimental results show that in the cross-day wet-to-wet transfer, the ALPHA performance is comparable to that of the wet-TRCA. However, in the cross-day wet-to-dry transfer, the ALPHA performance is significantly better than that of the dry-TRCA, and a high-speed zero-training SSVEP–BCI can be achieved with less training burden. The proposed transfer learning-based scheme could facilitate the long-term usage of the SSVEP–BCI and could also promote the implementation of the dry electrode-based SSVEP–BCI in real-world applications.

## Data Availability Statement

The data that support the findings of this study are available upon reasonable request from the authors.

## Ethics Statement

The studies involving human participants were reviewed and approved by the institutional review board of Tsinghua University. The patients/participants provided their written informed consent to participate in this study.

## Author Contributions

XL performed the data collection. XL and BL conducted the analysis and wrote the manuscript. GD and XG revised the manuscript. YW supervised the study. All authors contributed to the article and agreed to the published version of the manuscript.

## Conflict of Interest

The authors declare that the research was conducted in the absence of any commercial or financial relationships that could be construed as a potential conflict of interest.

## Publisher’s Note

All claims expressed in this article are solely those of the authors and do not necessarily represent those of their affiliated organizations, or those of the publisher, the editors and the reviewers. Any product that may be evaluated in this article, or claim that may be made by its manufacturer, is not guaranteed or endorsed by the publisher.
