# Supplementary material for: Facilitating Applications of SSVEP-Based BCIs by Within-Subject Information Transfer
Source: Front Neurosci. 2022 May 26;16:863359. doi: 10.3389/fnins.2022.863359 (PMC9198902; doi:10.3389/fnins.2022.863359)
Supplement: Supplementary file 1 [file Data_Sheet_1.pdf]

Supplementary Material

As for the cross-day wet-to-dry transfer, we conducted a series of feature visualization before and after ALPHA. The Accuracy and ITR of the spatially filtered test trials in feature space were compared before and after ALPHA, as illustrated in **Figure 1**. The average result of sixteen subjects showed that ALPHA significantly boosted the Accuracy and ITR of the spatially filtered test trials at all data lengths ($p < 0.05$). The feature space before the correlation operation was assessed by t-SNE(Van der Maaten and Hinton, 2008). The resultant low-dimensional manifold was compared before and after the subspace alignment of the AC (align covariance) module in ALPHA, as illustrated in **Figure 2A** and **2B**. Each subfigure illustrates the cross-day-cross-electrode data from a subject, and each dot represents a trial. The spatially filtered test trials (red dots), spatially filtered SSVEP template with ALPHA (green dots), and spatially filtered SSVEP template without ALPHA (blue dots) were illustrated. After employing ALPHA, it is noticeable that the SSVEP templates were moved toward the test trials in the t-SNE feature space. The result indicates the ALPHA (the AC module) can help reduce the distance between the SSVEP template and the test trial by domain adaptation in the feature space before the correlation operation. The feature space before and after the ASP (align spatial pattern) module in ALPHA was illustrated in **Figure 2C** and **2D**. Each subfigure illustrates the cross-day-cross-electrode data from a subject, and each dot represents a trial. The spatially filtered SSVEP template (red dots), spatially filtered test trials with ALPHA (green dots), and spatially filtered test trials without ALPHA (blue dots) were illustrated. Here each cluster formed by the dots represents a class. Since the SSVEP templates (wet-electrode EEG) have a high SNR and the templates are similar across cross-validation, the red dots in the cluster are dense. As for the ASP in ALPHA, it is noticeable that the test trials were moved toward the SSVEP templates in the t-SNE feature space. This further validates the efficacy of ALPHA (the ASP module) in reducing the distance in the feature space.

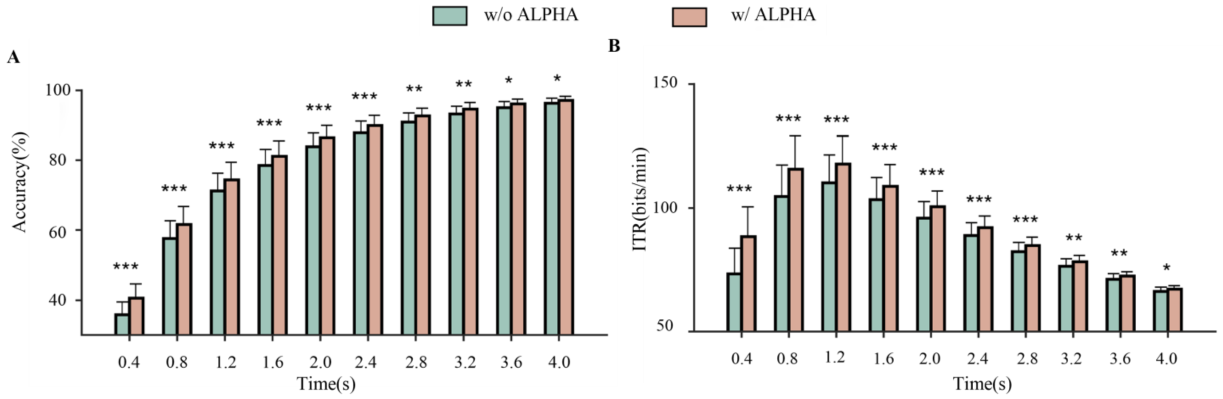

Supplementary Figure 1. The effect of ALPHA on the Accuracy (A) and ITR (B) in the feature space. Data lengths from 0.4s to 4s with an interval of 0.4s were evaluated. Here “w/o ALPHA” and “w/ ALPHA” denote the domain adaptation (subspace alignment) not applied and applied in ALPHA, respectively. The asterisks indicate a statistical significance ($^{*}p < 0.05$, $^{**}p < 0.01$, and $^{***}p < 0.001$).

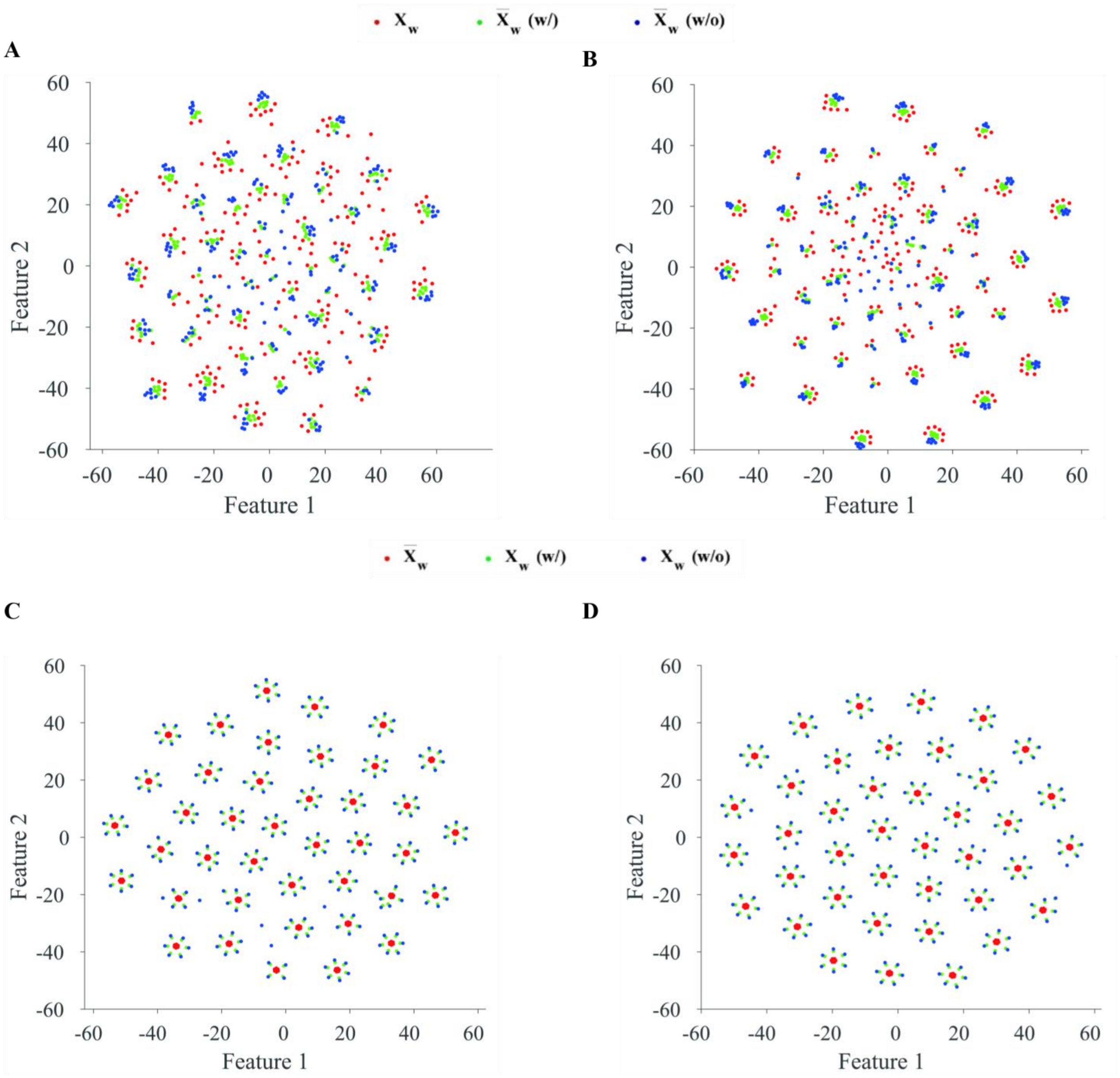

Supplementary Figure 2. The effect of ALPHA on the low dimensional feature space projected by t-SNE. The data length of 4s was used for evaluation. Here “w/o” and “w/” denote the domain adaptation (subspace alignment) not applied and applied. Here each subfigure illustrates the cross-day-cross-electrode data from a subject, and each dot represents a trial. **A** and **B** are for **AC** (red: spatially filtered test trials; green: spatially filtered SSVEP template with AC; blue: spatially filtered SSVEP template without AC); **C** and **D** are for **AS**P (red: spatially filtered SSVEP template; green: spatially filtered test trials with ASP; blue: spatially filtered test trials without ASP).

References

Van der Maaten, L., and Hinton, G. (2008). Visualizing data using t-SNE. *Journal of machine learning research* 9(11).
